# Notes and News

**Published:** 1896-04-11

**Authors:** 


### Notes and News.

(Collected and Communicated.)

### HOSPITAL MEETINGS.

H.R.H. the Duke of York has consented to preside at a festival dinner on behalf of the Royal Albert Orphan Asylum, Bagshot, at the Savoy Hotel, on May 21st.

Mr. George William Palmer, J.P. (of Reading), will preside at the festival dinner to be held at the Whitehall Rooms, Hotel Metropole, on Thursday, April 16th next, in aid of the Orphan Working School.

It is decided to erect a cottage hospital at Rothesay (N.B.) at a cost of £2,000.

Fifty thousand pounds is still required to complete the sum of £200,000 necessary to open the new Birmingham General Hospital free of debt.

It is proposed to erect a maternity hospital in Sydney. A building fund of £20,000 has been collected, and the Government is asked to grant the required land for the site.

The dedication service of the new chapel at the British Homes for Incurables was held last week. The Duchess of Teck and a distinguished congregation were present at the cermony. Bishop Barry officiated. The Duchess inspected the home afterwards.

Mr. George A. Richardson has been appointed superintendent of the Royal Sea-Bathing Infirmary at Margate. Mr. Richardson has held responsible positions at the General Hospital, Birmingham; Royal Hospital for Incurables, Putney Heath; National Hospital for the Paralysed and Epileptic, Queen Square; and at the Royal Free Hospital, London, embracing a period of upwards of twenty years.

Hospital Sunday at Glasgow this year has produced a total of £4,069, an increase of £428 over the collection of last year. We are glad to note an improvement on the very poor result of the collection in 1895, which we then commented upon, but the increase does not yet bring the total up to a sum worthy of so great a city as Glasgow, and the clergy there do not yet appear to have been fully roused to a sense of their responsibilities and opportunities.

The Schaw Memorial Convalescent Home, just erected, near Glasgow, has now been opened and presented to the city. Miss Schaw, who has erected the building in memory of her brother, entertained a large party at the institution at the end of last month to celebrate the opening. Accommodation is provided for fifty inmates of both sexes. The building is of greystone, with a tower in the centre. The rooms are large and lofty. The Royal Infirmary directors have the Schaw Home at their disposal.

A Chinese physician is about to commence practice at St. Louis, in the United States. He is a graduate of the Royal Medical College of Canton.

H.R.H. the Prince of Wales has fixed June 10th for the banquet in aid of Guy's Hospital, to be held under his presidency, at the Imperial Institute.

The Hospital of St. Sauveur, at Lille, was partially burnt last week, owing to the flames spreading from the neighbouring church of the same name, which was entirely destroyed. No patients were injured by the fire, though one expired through the shock attendant on removing. A shocking fatality occurred in connection with the fire. Fifteen soldiers, occupied in arresting the further progress of the flames, while removing the contents of the pharmacie drank a quantity of poison, which they imagined to be gin. Six died from its effects.

Mr. Sidney Phillips., B.A. Lond., late steward and assistant secretary at St. Mary's Hospital, has been appointed steward of St. Thomas's Hospital. The career of Mr. Phillips has been honourable and successful. In the beginning of 1889 Mr. Phillips was elected secretary of the Hospitals Association, in 1890 he became the chief clerk at St. Mary's Hospital, and some two years afterwards he was promoted to the office of steward of that institution, being elected assistant secretary in June, 1895. During Mr. Phillips' tenure of office at St. Mary's the expenditure at the institution has been very materially reduced. He has acquired an exceptional insight into the multifarious duties attaching to the office of steward to a large institution, is a competent bookkeeper and accountant, and has a sound knowledge of hospital administration. We congratulate Mr. Phillips upon his appointment at St. Thomas's, and believe that the institution will greatly benefit by his faithful and experienced services.

The election of Dr. Wilks to the presidency of the Royal College of Physicians is an event on which we may congratulate the Fellows of that ancient corporation. They have taken what, for the sake of their College, was clearly the right course, and they have conferred honour where honour certainly was due. Not only was Dr. Wilks peculiarly fitted for the post, as we pointed out a few weeks ago, but it was high time that Guy's Hospital should be represented on the list of presidents. We need not hesitate to take to ourselves some share in the credit of the happy and almost unanimous choice which has fallen on Dr. Wilks. Till a fortnight before the date of the election his name had been put on one side, and the choice was supposed to lie between others—good men, but not, as we thought, the most appropriate. So important did the matter seem to us that on March 21st we devoted a leading article to showing for how many reasons it was desirable that Dr. Wilks should be elected, and we took such measures as we thought necessary to bring those reasons prominently before the Fellows of the College, with the result that on March 30th Dr. Wilks was elected by the enormous majority of 114 against 22. We may fairly hope that those who profess to doubt how far The Hospital can properly be called a medical journal will, after this, recognise that when we intervene in the highest medical affairs we do so with effect; and also that we are so thoroughly in sympathy with the feelings of the medical profession at large, that when we do intervene our recommendations are likely to be endorsed by them.

